# A Long‐Term Human Liver Spheroid Model for Assessing Silencing and Durability of GalNAc‐Conjugated siRNAs

**DOI:** 10.1111/cts.70536

**Published:** 2026-04-08

**Authors:** Gijs‐Jan Scholten, Clara Grundmann, Åsa Nordling, Caroline Coskun, Volker Engelhardt, Lingheswar Sadhasivam, Tomaž Einfalt, Magnus Ingelman‐Sundberg, Sander van Riet

**Affiliations:** ^1^ Department of Physiology and Pharmacology Karolinska Institutet Stockholm Sweden; ^2^ NOVARTIS Pharma AG Basel Switzerland

**Keywords:** asialoglycoprotein receptor, liver spheroids, silencing efficiency, siRNA stability

## Abstract

Advances in RNA interference technology have established it as a powerful therapeutic tool with important future potential. The design and the chemical modifications of the siRNA nucleotide backbone have greatly enhanced stability, durability, and pharmacokinetics while minimizing tolerability risks. The optimal combination of these modifications depends on the target gene, tissue, and RNA sequence, necessitating an iterative, experimental approach that currently relies heavily on animal models. To reduce the reliance and number of (humanized) animals required, we developed a human long‐term liver 3D spheroid model designed for screening GalNAc‐conjugated siRNAs which captures the process of uptake, potency, and durability for early in vitro screening. These liver spheroids remain viable in culture for at least 5 weeks while maintaining expression of the asialoglycoprotein receptor to facilitate GalNAc mediated uptake. siRNA was efficiently internalized by the spheroids without the need for transfection reagents, and its durable silencing efficiency was assessed by monitoring *AHSA1* target gene expression over time. Target gene silencing in the spheroid model persisted up to 5 weeks post‐treatment. Fluorescently labeled siRNA enabled visualization of uptake and distribution within the spheroid, revealing somewhat reduced siRNA accumulation in pericentral CYP3A4+ hepatocytes accompanied with somewhat reduced ASGR1 expression. No signs of hepatotoxicity were observed under the conditions used. By varying the number of phosphorothioate modifications in the siRNA backbone, distinct differences in silencing efficiency and durability were observed which were principally similar as obtained in vivo in mice. We propose that this long‐term human liver spheroid model provides a valuable preclinical platform for evaluating siRNA‐based therapeutics with respect to uptake, durability, and silencing efficiency, and could refine early in vitro screening and accelerate drug development.

## Study Highlights

- What is the current knowledge on the topic?
  ○ Chemical modifications to the siRNA backbone are essential for enhancing molecular stability, reducing immunogenicity, increasing resistance to enzymatic degradation, and minimizing toxicity.
  ○ Such modifications are pivotal for optimizing siRNA performance in therapeutic settings.
  ○ However, despite growing interest in siRNA‐based therapeutics, no long‐term in vitro model exists to evaluate silencing efficiency and durability.
  ○ As a result, preclinical development still relies heavily on humanized mouse models.
- What question did this study address?
  ○ Can the human liver spheroids be used as an in vitro model to evaluate the effect of backbone modifications of the GalNAc‐conjugated siRNA on the silencing efficiency and durability?
- What does this study add to our knowledge?
  ○ We present a new human spheroid model for assignment of the properties of structurally different GalNAc‐conjugated siRNA molecules.
  ○ The spheroids were maintained in culture for up to 5 weeks while retaining expression of the asialoglycoprotein receptor.
  ○ These spheroids can be directly treated with GalNAc‐conjugated siRNA without the need for transfection reagents, enabling sustained target gene silencing to be monitored over the entire five‐week period.
  ○ Using fluorescently labeled siRNA, intracellular trafficking was visualized, revealing heterogeneity in hepatocyte uptake capacity.
  ○ Moreover, the model enabled clear discrimination of silencing performance across five distinct siRNA backbones.
- How might this change clinical pharmacology or translational science?
  ○ This study shows that human liver spheroids provide a robust, scalable platform for evaluating GalNAc‐conjugated siRNA therapeutics.
  ○ The model offers a valuable tool for early‐stage compound selection, potentially accelerating screening workflows and reducing reliance on animal models in preclinical development.

## 1 Introduction

Advancements in the development of RNA interference (RNAi) technology have opened the way for a potent new class of therapeutic agents [1]. This is exemplified by regulatory approval and clinical use of small interference RNA (siRNA) drugs like patisiran and inclisiran, with numerous others in phase 2 and 3 clinical trials [2, 3, 4]. The process of RNAi utilizes a highly conserved natural mechanism which loads the siRNA duplexes into the RNA‐induced silencing complex (RISC), which retains the antisense strand and discards the sense strand. The RISC, which contains the catalytic protein Argonaute‐2 (Ago2), recognizes and binds to the full‐length target mRNA sequence, leading to cleavage and subsequent translational silencing [5]. This gene therapy approach enables precise targeting of disease‐causing genes, allowing for highly specific and potentially personalized treatments. The siRNA sequence designed to recognize the target mRNA can be meticulously optimized and validated, minimizing the risk of off‐target effects. In contrast, small molecules and antibodies often target protein structures with less specificity, increasing the likelihood of unintended interactions.

A major challenge in RNAi‐based therapeutics is the enhancement of the pharmacokinetic stability of siRNA by increasing its resistance to degradation by lysosomes, endo‐ and exonucleases. This is achieved by chemically modifying the siRNA backbone. Frequently used modifications target the 2′‐position of the ribose, such as 2′‐O‐methyl (2′OMe) and 2′‐deoxy‐2′‐fluoro (2′F), and the insertion of phosphorothioate (PS) linkages at the 3′ and 5′ termini, by replacing a nonbridging oxygen in the phosphodiester bonds with sulfur to enhance stability and resistance to degradation [6, 7].

Hepatocytes serve as an attractive target for siRNA agents due to their expression of the asialoglycoprotein receptor (ASGPR). The ASGPR is a membrane‐bound receptor primarily expressed on hepatocytes, which binds glycoproteins containing terminal galactose or N‐acetylgalactosamine (GalNAc) residues. Upon binding, ASGPR mediates endocytosis of these glycoproteins, leading to their eventual localization within the lysosomal compartment [8]. To enhance targeted delivery, a trivalent GalNAc moiety can be conjugated to the sense strand of the siRNA, facilitating efficient and specific hepatocyte uptake. During lysosomal maturation, the decreasing pH triggers the dissociation of ASGPR from the GalNAc‐conjugated siRNA, allowing the receptor to recycle back to the cell membrane [9]. The retained siRNA duplexes then undergo the incompletely understood process of endosomal escape to reach the cytoplasm, where they can be loaded onto the RISC. There is yet no universally optimal small interfering RNA backbone chemistry, as this depends on multiple factors, including the required pharmacokinetics, stability, binding properties, target gene, tissue, and the specific siRNA sequence [10, 11, 12]. In order to study the durability of siRNA backbone variations, reliable, long‐term human in vitro models are required, which are unfortunately lacking. Currently, siRNA design for a given therapeutic target remains, following a short‐term in vitro screen, a heavily animal model focussed process. The development and optimization of human therapeutic siRNAs requires humanized transgenic or AAV mice that carry the human transcript the siRNA is targeting. Due to high expense, developability and in vivo resources required for these models our aim was to develop a long‐term human in vitro model to refine the early in vitro screening. Thus, here we present a long‐term in vitro spheroid model composed of primary human hepatocytes, which enables the evaluation of the entire process of GalNAc mediated‐siRNA uptake, potency and durability. This human liver spheroid model has been extensively characterized and exhibits a phenotype closely resembling that of the in vivo human liver, as evidenced by transcriptomic, proteomic, and metabolomic profiling [13, 14]. Additionally, it has been employed to investigate liver pathologies such as steatosis, cholestasis, and fibrosis [15, 16, 17]. Using this model, we demonstrate that GalNAc‐mediated uptake in primary human hepatocytes can be monitored dynamically. Fluorescently labeled siRNA reveals selective intracellular distribution, with preferential accumulation in pericentral hepatocytes. Furthermore, we show that hepatic spheroids reveal differences in the silencing efficiency of various siRNA backbone modifications. These findings suggest that the human‐based spheroid model could serve as a valuable siRNA screening platform that could filter out candidates that have poor uptake or durability, offering the potential to reduce the reliance on, and number of, animals required.

## 2 Methods

### 2.1 Spheroids Culture

Primary human hepatocytes (PHH) were purchased from LONZA (Basel, Switzerland) or BioIVT (Westbury, NY, USA). The donor characteristics and Supporting Information are presented in Table 1. Spheroids were cultured as previously described [18]. PHH (1500 cells) were seeded into 96‐well Corning Costar Ultra‐Low Attachment Plates (Merck, Kenilworth, NY, USA) or in 96‐Well Nunclon Sphera U‐Shaped‐Bottom Microplate (Thermo Fisher, Waltham, MA, USA) and centrifuged at 130× *g* for 3 min. Spheroids were cultured in William's E medium supplemented with 2 mM L‐glutamine (Sigma‐Aldrich, Saint Louis, MO, USA), 100 U/mL penicillin (Sigma‐Aldrich), 100 μg/mL streptomycin (Sigma‐Aldrich), 100 nM dexamethasone (Sigma‐Aldrich), ITS X‐100 (Thermo Fisher), and 10% fetal bovine serum (FBS; Thermo Fisher). Following spheroid formation (~5 days), the cells were refreshed every 2–3 days with complete William's E medium but with 3% FBS. Spheroids were cultured in 100 μL of medium under standard cell culture conditions at 37°C in a humidified incubator with 5% CO_2_.

**TABLE 1:** Primary human hepatocyte donor characteristics.

Company	Cat. Nu./batch	Age	Sex	Ethnicity	BMI	Alcohol	Smoking	Drug	Diabetes
BioIVT	M00995‐P/CDP	58	Male	Caucasian	25.7	No	No	No	Yes
BioIVT	M00995‐P/GID	58	Male	African American	34.4	Minor	40 years	No	Yes
BioIVT	F00995‐P/IIN	57	Female	Caucasian	21.3	Minor	No	Opiates	No
Lonza	HUM182411	5	Male	Hispanic	19.3	No	No	No	No
Lonza	HUM191651	1	Male	Caucasian	17.8	No	No	No	Yes
Lonza	HUM183061	54	Male	Caucasian	29.0	Heavy	No	No	No
Lonza	HUM190131	48	Female	Caucasian	22.4	Heavy	Yes	No	Yes

### 2.2 HepG2 Culture

HepG2 cells (ATCC, Manassas, VA, USA) were cultured in T‐75 flasks (Thermo Fisher) and cultured in DMEM/F12 supplemented with 10% FBS. The cells were refreshed twice weekly and passaged every week. The cells were maintained under standard cell culture conditions at 37°C in a humidified incubator with 5% CO_2_.

### 2.3 siRNA Treatment

Additional information about the siRNA variants and GalNAc‐conjugate (Axolabs, Kulmbach, Germany) can be found in Table 2 and Figure S1A. For free uptake experiments GalNAc‐conjugated siRNA was directly added to the cultures in the desired concentration. For control transfections or for siRNA lacking GalNAc, Lipofectamine RNAiMax (Thermo Fisher) was used according to manufacturer's instructions. siRNA was diluted in OptiMEM (Thermo Fisher) to the desired concentration. Additionally, Lipofectamine RNAiMAX was diluted in OptiMEM to a final volume of 0.2 μL per well. Following a 5‐min incubation, siRNA and lipofectamine was mixed in a 1:1 ratio. The mixture was then incubated at room temperature for 20 min and added to the cultures. For distribution and trafficking experiments GalNAc‐conjugated Cy3 fluorescently labeled siRNA was added for free uptake on day 0 or day 7.

**TABLE 2:** siRNA sequences and backbone structures.

Compound	Strand	Sequence 5′ to 3′	No. of PS linkage/strand	Total no. of PS linkage
Parent	AS	fsUfsuCfaUfuAfaGfgCfcAfcGfaGfasusu	4	6
	S	uscsUfcGfuGfgCfcUfuAfaUfgAfaAf(GalNAc)	2	
Sense w/o PS	AS	UfsUfsuCfaUfuAfaGfgCfcAfcGfaGfasusu	4	4
	S	ucUfcGfuGfgCfcUfuAfaUfgAfaAf(GalNAc)	0	
3′ w/o PS	AS	UfsUfsuCfaUfuAfaGfgCfcAfcGfaGfauu	2	4
	S	uscsUfcGfuGfgCfcUfuAfaUfgAfaAf(GalNAc)	2	
5′ w/o PS	AS	UfUfuCfaUfuAfaGfgCfcAfcGfaGfasusu	2	4
	S	uscsUfcGfuGfgCfcUfuAfaUfgAfaAf(GalNAc)	2	
3′ + 5′ w/o PS	AS	UfUfuCfaUfuAfaGfgCfcAfcGfaGfauu	0	2
	S	uscsUfcGfuGfgCfcUfuAfaUfgAfaAf(GalNAc)	2	
Parent w/o GalNAc	AS	UfsUfsuCfaUfuAfaGfgCfcAfcGfaGfasusu	4	6
	S	uscsUfcGfuGfgCfcUfuAfaUfgAfaAf	2	
Positive control metabolic stability	AS	UfsUfsuCfaUfuAfaGfgCfcAfcGfaGfasusu	4	7
	S	uscsUfcGfuGfgCfcUfuAfaUfgAfaAfs(GAlNAc)	3	

### 2.4 Poly‐l‐Lysine

To stain single cells, they were first adhered to immunohistochemistry adhesive slides (Histolab, Askim, Sweden) using a poly‐L‐lysine coating. To coat, 50 μL droplets of 0.01% (*w*/*v*) poly‐L‐lysine (Sigma‐Aldrich) were left at room temperature until evaporated. Cells were collected in 50‐μL medium containing 10% FBS and placed on the poly‐L‐lysine‐coated area until all the liquid evaporated. After evaporation, the cells were fixed with 4% paraformaldehyde and stained, as described below.

### 2.5 Cell Viability

Cell viability was measured using the CellTiter Glo Luminescent Cell Viability Assay kit (Promega, Madison, WI, USA) according to the manufacturer's instructions. First, 80 μL of the total of 100 μL medium was removed from each well, and 25 μL of reconstituted assay reagent was added. The spheroids were then incubated for 20 min in the dark at 37°C. The luminescence signal was measured using a MicroBeta LumiJET 2460 Microplate Counter (Perkin Elmer, Waltham, MA, USA) in white flat‐bottom 96‐well plates. For each timepoint, at least 4 spheroids were measured per condition.

### 2.6 Immunofluorescent Staining

The spheroids were fixed for 2 h at room temperature in 4% paraformaldehyde and subsequently dehydrated in 30% sucrose for at least 2 days at 4°C. The spheroids were embedded and frozen using Tissue‐Tek OCT (Sakura Finetek, Alphen aan den Rijn, The Netherlands) and sectioned at 8 μm using a NX70 Cryostat (Thermo Fisher). Spheroid sections were blocked using 1% BSA and 0.25% Triton X‐100 in PBS (PBST, Sigma‐Aldrich) for 2 h at room temperature.

The spheroid sections were stained with a primary antibody diluted in PBST overnight at 4°C. A list of the antibodies is provided in Table 3. Next, the slides were washed 3 times with PBS. The secondary antibody diluted in PBST was added and incubated for 2 h at room temperature in the dark. The slides were again washed 3 times with PBS. The slides were mounted with ProLong Gold Antifade containing DAPI (Thermo Fisher) and imaged using an Olympus IX73 inverted microscope (Olympus, Tokyo, Japan) and LSM800‐Airy Zeiss (Carl Zeiss AG, Oberkochen, Germany). Images were analyzed using the Fiji software version 2.12.0.

**TABLE 3:** Antibodies.

Antibody	Species	Company	Cat. No.	Dilution
Primary antibodies				
ASGR1	Rabbit	Thermo Fisher	PA5‐32030	1:100
CYP3A4	Rabbit	CYPEX	PAP011	1:5000
Phalloidin	488‐conjugated	Thermo Fisher	A12379	1:100
Secondary antibodies				
Alexa Fluor 488	Goat‐α‐Rabbit	Thermo Fisher	A11008	1:500

### 2.7 RNA Isolation, cDNA Synthesis, and qPCR

RNA isolation was performed using miRNeazy (Qiagen, Hilden, Germany) according to manufacturer's instruction. RNA concentration was determined using a Qubit 4 fluorometer (Thermo Fisher). cDNA was generated with SuperScript III reverse transcriptase (Thermo Fisher) using a SimpliAmp Thermal Cycler (Thermo Fisher) according to the manufacturer's protocol. Quantitative PCR reactions were performed using 2X TaqMan Universal PCR mix (Thermo Fisher) on a 7500 Fast Real‐Time PCR system (Applied Biosystems, Waltham, MA) with 20X TaqMan probes (Table 4). Gene expression was analyzed using the delta–delta Ct method (2−ΔΔCt), with TATA‐Box Binding Protein (TBP) as housekeeping gene.

**TABLE 4:** Taqman and stemloop primers.

Gene	TaqMan probe nr	Supplier
*ALB*	Hs00910225_m1	Thermo Fisher
*CYP3A4*	Hs00604506_m1	Thermo Fisher
*GAPDH*	Hs99999905_m1	Thermo Fisher
*TBP*	Hs00427620_m1	Thermo Fisher
*AHSA1*	Hs00201602_m1	Thermo Fisher
*ASGR1*	Hs01005019_m1	Thermo Fisher
Primer	Sequence	Supplier
Stem‐loop AHSA1 RT primer	GTCGTATCCAGTGCAGGGTCCGAGGTATTCGCACTGGATACGACAATCTCGTGG	IDT
AHSA Primer FWD	TCGTGATTTCATTAAGGCCAC	IDT
Stem‐loop Universal Primer REV	GTGCAGGGTCCGAGGT	IDT
Universal Taqman Probe	(6‐FAM)‐5′‐TTCGCACTGGATAC‐MGB‐3′	Thermo Fisher

### 2.8 Cellular siRNA Determination

Samples that were used to measure siRNA concentration were isolated using RNeasy Micro Kit (Qiagen) according to the manufacturer's instructions. To determine the concentration of siRNA present in spheroids, a stem‐loop qPCR was performed. Samples and standard were diluted in PBS with 0.25% Triton X‐100 (PBST; Thermo Fisher). Samples were diluted 100× in PBST. The samples and standard were first heated for 10 min at 95°C to denature the siRNA duplexes. Next, stem‐loop reverse transcription was performed using the microRNA Reverse Transcription kit (Thermo Fisher), using AHSA1 siRNA directed stem‐loop primer (IDT, Coralville, IA, USA). For reverse transcription in SimpliAmp thermal cycler (Thermo Fisher) the following program was used (30 min 16°C; 30 min 42°C; 5 min 85°C; 4°C). Subsequently, qPCR was performed using TaqMan Universal PCR Master Mix, no AmpErase UNG (Thermo Fisher) with 2.5 μM forward primer (IDT), 1.17 μM reverse primer (IDT), and 0.33 μM universal TaqmanTM probe (Thermo Fisher). Primer sequences are listed in Table 4. The plate was then run on a Step One Plus Real‐Time PCR (2 min 50°C; 10 min 95°C; 40 cycles of 15 s 95°C, 1 min 55°C). Sample siRNA concentrations were extrapolated from the siRNA dilution series and corrected for dilution factor.

### 2.9 Metabolic Stability Assay

siRNAs were diluted in rat liver tritosomes (2.5 mg/mL; BioIVT) to a final concentration of 6 μM. For assay preparation, 10 μL of 60 μM siRNA was mixed with 90 μL of tritosomes in a skirted 96‐well PCR plate (250 μL; Eppendorf). Samples were incubated at 37°C (MaxQ 6000 shaker) for 0, 8, or 72 h. At each timepoint, 100‐μL aliquots were collected, mixed with Clarity OTX lysis buffer (Phenomenex, Torrance, CA), and stored at −80°C. The *t* = 0 control was prepared by repeating the initial dilution immediately before extraction. For solid‐phase extraction, 150 μL of sample was applied to Clarity OTX 96‐well plates (Phenomenex) preconditioned with 150 μL methanol (Sigma‐Aldrich) at 5–6 psi and equilibrated with 150 μL of 50 mM ammonium acetate (Sigma‐Aldrich, pH 5.5). Wells were washed with 150 μL of 50 mM ammonium acetate in 50:50 water: acetonitrile (Sigma‐Aldrich, pH 5.5) at 6.5–7.5 psi and eluted twice with 50 μL of buffer containing 100 mM ammonium bicarbonate, 40% acetonitrile, 10% tetrahydrofuran, and 10 mM TCEP (Sigma‐Aldrich, pH 9.4) at ≥ 7.5 psi.

UHPLC analysis was performed on a Thermo Vanquish system (Thermo Fisher) using a Biozen C18 column (1.7 μm, 2.1 × 50 mm; Phenomenex) at 75°C. The mobile phase consisted of 8.5 mM triethylamine (Sigma‐Aldrich) and 200 mM hexafluoroisopropanol (Sigma‐Aldrich) in water (A) and methanol (Sigma‐Aldrich) (B), with a 10%–45% B linear gradient over 4.5 min at 0.3 mL/min.

Mass spectrometry was conducted on a Q Exactive HF‐X Orbitrap (Thermo Fisher) under the following settings: 2.9 kV capillary voltage, 35 V sampling cone, 4 V extraction cone, 150°C source, 350°C desolvation, and 600 L/h desolvation gas flow.

### 2.10 In Vivo Treatment and Sample Preparation

The 8‐week‐old C57BL/6 female mice were obtained from Charles River Laboratories Germany. Ad libitum food and water were provided to the mice over the entire period. For evaluation, 3 mice per group and time point received PBS or siRNA by subcutaneous injection (s.c. bolus) with a nominal administration volume of 5 mL/kg, at a nominal dose of 25 and 50 mg/kg. After 168 h (7 days) post‐treatment, the mice were anesthetized with isoflurane. The euthanasia was performed via heart section, and the liver was harvested under anesthesia. The liver samples were weighed and collected in homogenization tubes (Omni International, Kennesaw, GA, USA) and frozen at −80°C until processing. For homogenization and lysis, four volumes of Trizol reagent (Thermo Fisher) were added per one weight‐part of tissue (resulting in a 1:5 dilution of sample in Trizol). Homogenization was performed using the BeadRuptor Elite and BR Cryo‐device (Omni international) at 5°C. A total of 8 homogenization cycles were performed at 5 m/s for 30 s, with 20‐s breaks between each cycle. The homogenized samples were centrifuged for 1 min at 4000 rpm in new 1.5 mL PCR‐clean Eppendorf tubes. Only the supernatant was collected, frozen at −20°C, and used for siRNA quantification.

RNA isolation was performed using the RNeasy Mini Kit (Qiagen) on the QIAcube (Qiagen), following the manufacturer's instructions. RNA concentration was determined using a Nanodrop fluorometer (Thermo Fisher). Samples were diluted to a final concentration of 5 ng/μL in nuclease‐free water (Roche, Basel, Switzerland). One‐step RT‐qPCR was performed using the QuantiTect Multiplex RT‐PCR NR Kit (Qiagen) and 20X TaqMan primer/probe (Table 4). Two μL of sample was added to 8 μL of mastermix. The plate was run on a QuantStudio 7 flex Real‐Time PCR System (Thermo Fisher) using the following program: 2 min 50°C; 10 min 95°C; 40 cycles of 15 s 95°C and 1 min 60°C. Gene expression was analyzed using the delta–delta Ct method (2−ΔΔCt), with PPIB as the reference gene.

### 2.11 Statistical Analysis

Statistical analysis was performed using GraphPad Prism version 10.1.2 (GraphPad Software, San Diego, CA, USA), and graphs are shown as the mean and SEM. Statistical analysis of differences was performed using a paired two‐sided *t*‐test and comparing each condition to the appropriate control, one‐ or two‐way ANOVA. *p < 0.05; **p < 0.01; ***p < 0.01; ****p < 0.01.

### 2.12 Ethics Statement

All research was conducted in accordance with both the Declarations of Helsinki and Istanbul, all research was approved by the appropriate ethics and/or institutional review committee(s), and written consent was given in writing by all subjects. PHH were obtained from commercially available sources and required no ethical approval from the Karolinska Institute. Copies of documentation from Lonza and BioIVT regarding the written consent of donors were obtained from the respective companies. Liver tissue samples were obtained from the Huddinge Liver Bank and approved by the Regional Ethical Committee (D: nr 2017/269‐31).

Novartis is committed to conducting in vivo research only when scientifically justified and in full compliance with applicable laws and ethical standards, guided by the 3Rs (Replace, Reduce, Refine), rigorous independent review, animal welfare committee review, qualified personnel, principles of humane care and endpoints, and transparency in reporting, and continuous improvement, ensuring animal welfare and scientific integrity remain central to the development of safe and effective medicines. Procedures involving animals were reviewed and approved by the cantonal veterinary authority of Basel‐Stadt, Switzerland (license BS‐1587).

## 3 Results

### 3.1 Characterization of Long‐Term Human Liver Spheroid Cultures

Evaluating the silencing efficiency of siRNA constructs and their backbone modifications in vitro with suitable predictive accuracy requires a stable, long‐term human model. Hepatic spheroids derived from PHH provide a promising solution for this purpose. These spheroids can be cultured for at least 5 weeks while maintaining structural integrity, stable expression of liver‐specific markers, and functional activity [13].

**FIGURE 1:**
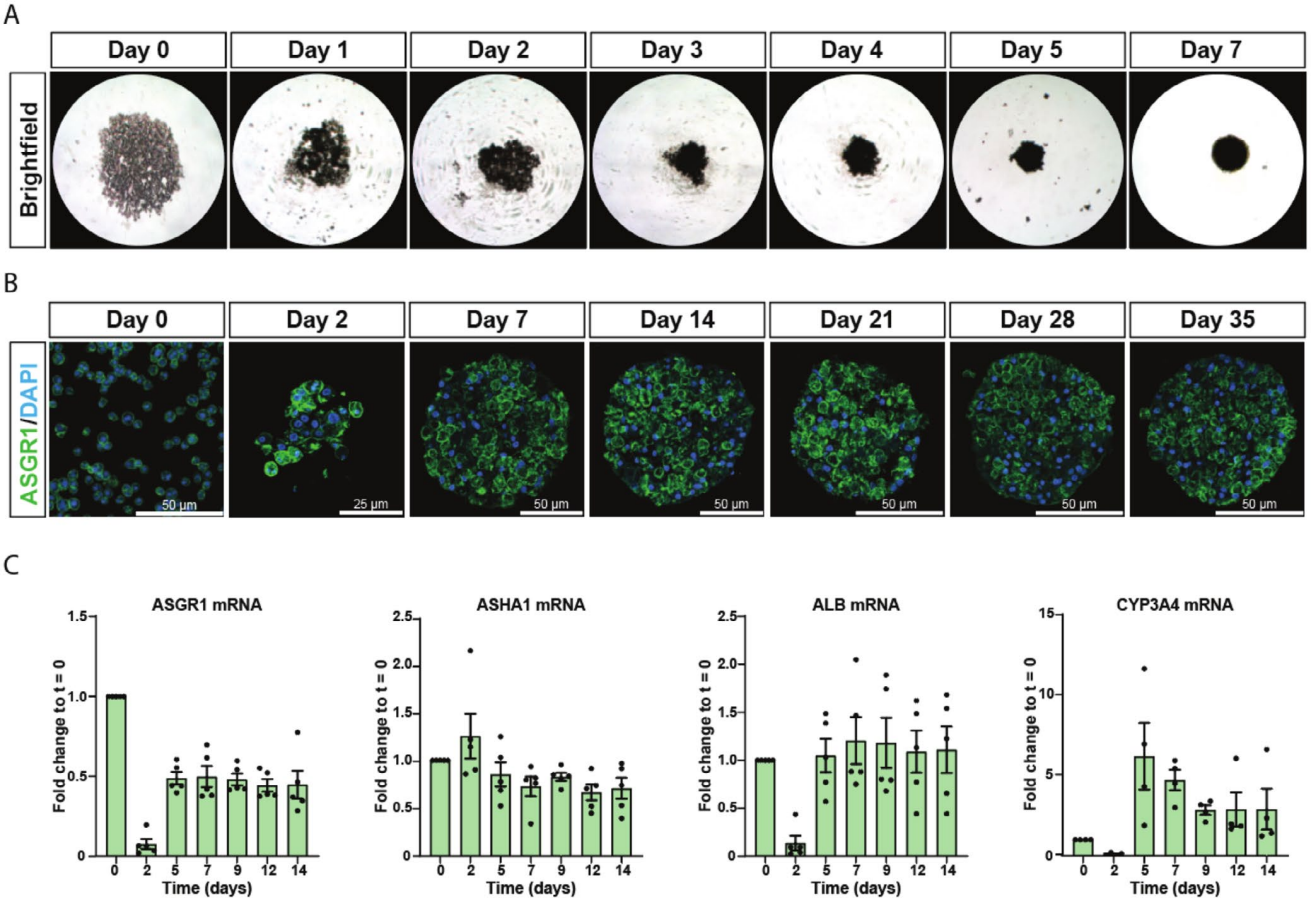
Characterization and long‐term culture of liver spheroids. (A) Representative brightfield images of primary human hepatocytes aggregating into a spheroid over 7 days. (B) Fluorescent images of primary human hepatocytes at different stages of spheroid formation and culture, stained for ASGR1 (green) and nuclei (blue). (C) Analysis of mRNA expression of ASGR1, AHSA1, Albumin and CYP3A4 in spheroids at different times over 14 days. (*n* = 4–5 independent experiments). ALB, albumin; ASGR1, asiologlycoprotein receptor 1; AHSA1, activator of 90 kDa heat shock protein ATPase homolog 1; CYP3A4, cytochrome P450 3A4.

To generate hepatic spheroids, PHH are seeded into ultra‐low attachment plates, where they spontaneously aggregate into dense three‐dimensional structures over 5–7 days (Figure 1A). The siRNA constructs used in this study are conjugated to a trivalent N‐acetylgalactosamine (GalNAc) moiety at the 3′ end of the sense strand. This GalNAc modification facilitates targeted and rapid internalization via endocytosis by binding to the asialoglycoprotein receptor (ASGPR) on the hepatocyte surface.

The primary ASGPR subunit, ASGR1, was detected and remained consistently expressed on the surface of PHH throughout the five‐week culture period (Figure 1B). Initially, ASGR1 expression is homogeneous across cells (Day 0), but following spheroid formation, distinct subpopulations of high and low ASGR1‐expressing cells emerge. During spheroid formation, mRNA expression of ASGR1, along with the hepatocyte‐associated markers albumin and cytochrome P450 3A4 (CYP3A4), initially declines but subsequently increases following spheroid aggregation when the cells are re‐differentiated (Figure 1C). Thus, the previously described process of hepatocyte de‐differentiation upon dissociation is observed, followed by redifferentiation during spheroid formation, driven by the loss and subsequent reestablishment of cell–cell interactions [14, 19].

Furthermore, the expression of Activator of HSP90 ATPase Activity 1 (AHSA1), the target gene in this study, remains stable for the first 2 weeks of culture (Figure 1C), supporting its suitability for long‐term siRNA evaluation.

### 3.2 Treatment of Hepatic Spheroids With Chemically Modified GalNAc‐Conjugated siRNA

**FIGURE 2:**
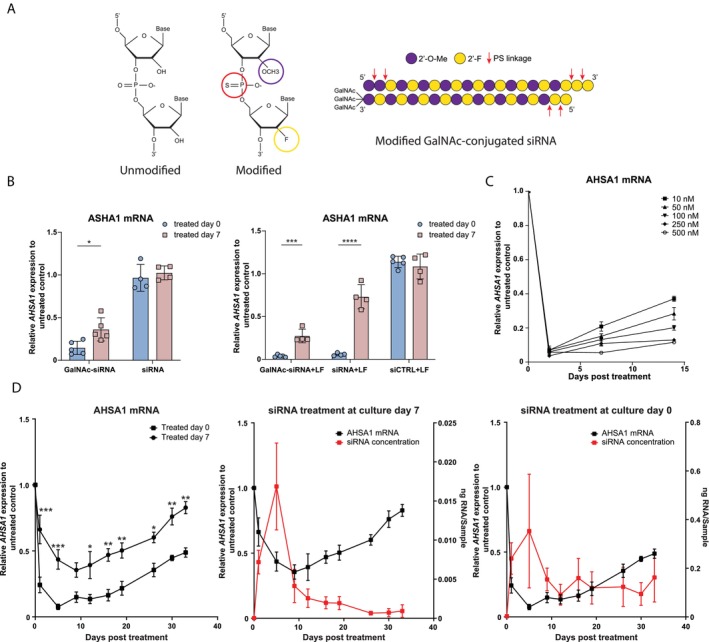
Treatment of liver spheroids with GalNAc‐conjugated siRNA. (A) Structures of unmodified and 2′‐O‐methyl (2′OMe) and 2′‐deoxy‐2′‐fluoro (2′F) modified nucleotides and an overview of the GalNAc‐conjugated siRNA backbones. (B) *ASHA1* gene expression in liver spheroids 7 days post‐treatment following unconjugated or GalNAc‐conjugated siRNA (150 nM, free uptake) treatment on day 0 (prior to spheroid formation) or day 7 (after spheroid formation) (left) (*n* = 4–5 independent experiments), lipofectamine (LF) mediated transfection with unconjugated, GalNAc‐conjugated or scrambled siRNA (150 nM) treatment on day 0 (prior to spheroid formation) or day 7 (after spheroid formation) (right) (*n* = 4–5 independent experiments). (C) *AHSA1* gene expression on day 2, 7 and 14 post‐treatment with different concentrations of GalNAc‐conjugated siRNA (free uptake) (*n* = 3 independent experiments). (D) (left panel) overlay of *AHSA1* gene expression post‐treatment in long‐term cultures of liver spheroids treated with GalNAc‐conjugated siRNA (150 nM, free uptake) on day 0 (prior to spheroid formation) or day 7 (after spheroid formation). (Middle panel) Overlay of *AHSA1* gene expression and corresponding siRNA concentration post‐treatment in spheroid cultures treated on day 7. (Right panel) Overlay of *AHSA1* gene expression and corresponding siRNA concentration post‐treatment in spheroid cultures treated on day 0. *p < 0.05; **p < 0.01; ***p < 0.001; ****p < 0.0001.

To evaluate the suitability of hepatic spheroids as an in vitro model for studying siRNA silencing efficiency, we first utilized an siRNA construct with commonly employed modifications. This standard (parent) siRNA includes a GalNAc moiety (Figure S1A), alternating 2′‐OMe and 2′‐F modified bases, and two phosphorothioate (PS) linkages at both the 3′ and 5′ prime ends of the antisense strand, along with two PS linkages at the 5′ prime end of the sense strand (Figure 2A).

First, we assessed whether the GalNAc moiety facilitated endocytosis of the siRNA into hepatocytes (free uptake) and compared this to lipofectamine‐mediated uptake. Second, we investigated whether the timing of treatment, either day 0 (prior to spheroid formation) or day 7 (after spheroid formation), influenced the extent of gene silencing. Cultures were treated with 150 nM siRNA on either day 0 (prior spheroid formation) or day 7 (after spheroid formation) and analyzed 7 days post‐treatment (Figure 2B). AHSA1 mRNA expression was significantly decreased in both day 0 (14.6% ± 6.9%) and day 7 (36.2% ± 12.1%) treated samples. In contrast, no silencing was observed in cultures treated with siRNA lacking the GalNAc moiety, indicating that GalNAc facilitates siRNA internalization via ASGPR binding (Figure 2B).

To determine whether free uptake is rate‐limiting, we repeated the experiment using lipofectamine‐mediated transfection. Transfection on day 0 (prior to spheroid formation) resulted in high levels of silencing for both GalNAc‐conjugated (3.8% ± 1.4%) and non‐conjugated siRNA (5.8% ± 1.6%). Although lipofectamine‐mediated transfection of siRNA on day 7 (after spheroid formation) resulted in AHSA1 silencing, the effect was noticeably less compared to transfection on day 0. Furthermore, transfection on day 7 (after spheroid formation) with GalNAc‐conjugated siRNA (27.3% ± 7.9%) was more effective compared to non‐conjugated siRNA (73.2% ± 14.1%) (Figure 2B).

To determine siRNA concentration range, cultures were exposed to varying GalNAc‐siRNA concentrations on day 0 (prior spheroid formation), and AHSA1 expression was assessed 2‐, 7‐, and 14‐days post‐treatment (Figure 2C). Remarkably, all concentrations exhibited strong and comparable silencing by day 2 post‐treatment, followed by a recovery of AHSA1 expression in a concentration‐dependent manner. Indicating a strong initial potency of this siRNA variant and concentration dependent durability. Spheroid viability remained unaffected, even at higher siRNA concentrations (Figure S1B).

Next, cultures were treated with siRNA either on day 0 (prior to spheroid formation) or on day 7 (after spheroid formation) of culture, and AHSA1 expression was monitored for 33 days post‐treatment (Figure 2D). In both conditions, AHSA1 expression was decreased, which persisted for 17–19 days, after which expression was gradually restored. Thirty‐three days post‐treatment, the spheroids treated on day 0 (prior to spheroid formation) showed 48.8% ± 5% residual expression relative to untreated controls, whereas spheroids treated on day 7 (after spheroid formation) showed 82.7% ± 8% residual expression. Importantly, there was no loss of viability compared to untreated controls (Figure S1C). Cellular siRNA levels showed an inverse relationship to AHSA1 expression, increasing rapidly during early time points and decreasing gradually afterwards. The decrease in intracellular siRNA is consistent with the decrease in silencing capacity. A similar pattern was observed when lipofectamine was used to transfect hepatocytes on day 0 (prior to spheroid formation), independent of GalNAc moiety (Figure S1D).

While siRNA was detectable throughout the culture in the day 0 condition, it was nearly undetectable in the day 7 treated condition after 25 days (Figure 2D).

### 3.3 Uptake and Distribution of siRNA in Liver Spheroids

**FIGURE 3:**
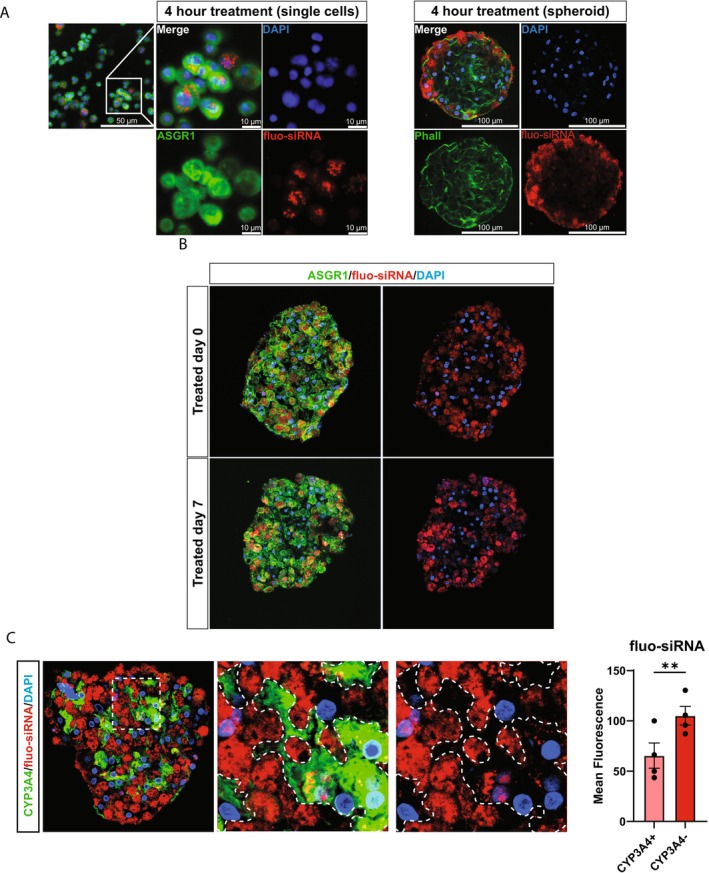
siRNA uptake and distribution. (A) Illustrative immunofluorescent images of primary human hepatocytes following 4‐h incubation with GalNAc‐conjugated fluorescent siRNA (fluo‐siRNA) (200 nM) on poly‐l‐lysine coated slides (left) and liver spheroid treated for 4 h with fluo‐siRNA (200 nM) cryosectioned and stained with phalloidin (green) (right). (B) Illustrative immunofluorescent images of cryosection liver spheroids 7 days post treatment with fluo‐siRNA (200 nM) (red) in comparison to ASGR1 expression (green). (C) Representative immunofluorescent image of liver spheroids treated with fluo‐siRNA (red) and stained for CYP3A4 (green). The mean fluorescent signal of the fluo‐siRNA was measured in the CYP3A4+ and CYP3A4− area of the spheroid (*n* = 4). **p < 0.01.

To study the uptake and distribution of siRNA within spheroids, a fluorescently labeled GalNAc‐conjugated siRNA (fluo‐siRNA) was used. Primary human hepatocytes in suspension and liver spheroids were treated with 200 nM fluo‐siRNA for 4 h. Following incubation, the spheroids were collected, and single cells were fixed onto poly‐L‐lysine‐coated slides, stained, and analyzed (Figure 3A). Analysis revealed that 94.9% ± 4.3% of the cells showed fluo‐siRNA positivity, with the fluorescent signal localized primarily in perinuclear foci, suggesting accumulation in endosomal structures. In contrast, fluo‐siRNA treatment of other liver cell types in suspension (crude fraction of non‐parenchymal cells) showed no detectable uptake after 4 h (data not shown). Within spheroids treated with fluo‐siRNA, the signal after 4 h was predominantly concentrated in the more outer cell layers. The fluorescence in these cells was intense, with individual foci difficult to distinguish, indicating an uneven distribution (Figure 3A).

To assess longer‐term distribution, spheroids were treated with fluo‐siRNA and analyzed after 7 days, with treatments administered at day 0 (prior to spheroid formation) or day 7 (after spheroid formation) (Figure 3B). The fluorescent signal in spheroids treated at day 0 or day 7 showed no clear difference in distribution throughout the spheroids. However, there were noticeable negative regions in both conditions. Further analysis of cellular distribution was performed by staining spheroids for CYP3A4, a marker of pericentral hepatocytes. This revealed a lower overall accumulation of fluorescent signal in CYP3A4+ cells compared to CYP3A4− cells within the spheroid (Figure 3C). This is in line with the immunohistochemical analyses of the ASGR1 distribution in human liver, where a higher grade of expression was seen in the perivenous area of the sinusoids having lower levels of CYP3A4 expression (Figure S2).

### 3.4 Screening of PS‐Modified siRNA Constructs

**FIGURE 4:**
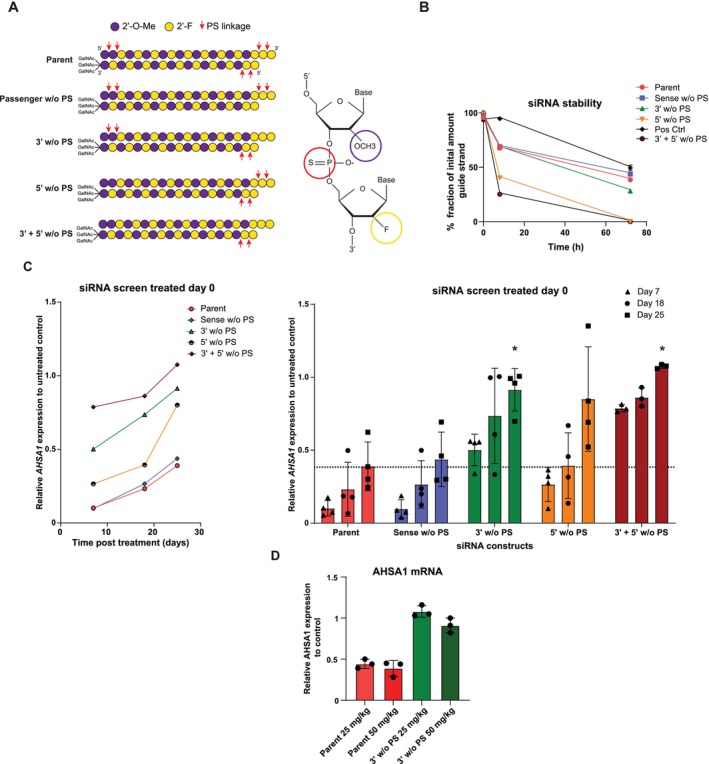
Screen of PS modified siRNA constructs. (A) Schematic overview of the different constructs with the phosphonothioate (PS) modifications. 2′F, 2′‐deoxy‐2′‐fluoro; 2′OMe, 2′‐O‐methyl; PS, phosphorothioate. (B) Chemical stability measurement of PS modified siRNA variants: removal of PS from the antisense strand reduced stability. Positive control for this assay is the parent construct with an extra PS modification on the 3′ of the sense strand. (C) Silencing efficiency of PS‐modified constructs in liver spheroid cultures treated on day 0 (100 nM, free uptake). AHSA1 expression was measured on day 7, 18 and 25 post‐treatment (left, mean values, right bar representation) (*n* = 4 independent experiments). (D) In vivo activity of parent and 3′ w/o PS variant siRNA. Mice were treated with either the parent construct or 3′ w/o PS variant at 25 or 50 mg/kg subcutaneously. Seven days post‐treatment, AHSA1 expression was measured (*n* = 3 animals per group).

We investigated whether hepatic spheroids were able to differentiate between silencing efficiency of five different siRNA constructs with distinct phosphorothioate (PS) backbone modifications. Each variant contained the same GalNAc moiety and alternating 2′‐OMe and 2′‐F modifications but differed in the positioning and amount of the PS modifications (Table 2 and Figure 4A).

The effect of PS modifications on construct stability was assessed (Figure 4B). Variants lacking PS modifications on the sense strand (Sense w/o PS), or on the 3′ end (3′ w/o PS), or at the 5′ end (5′ w/o PS) of the antisense strand were compared with the parent construct. Lack of PS modifications from the sense strand had no effect on stability. Removal from the antisense strand's 3′ end caused a modest stability reduction, whereas removal from the 5′ end resulted in a pronounced loss of stability. Showing a difference between 3′ to 5′ and 5′ to 3′ decay mechanisms [20].

To examine functional consequences, spheroid cultures were treated on day 0 (prior to spheroid formation) and AHSA1 silencing was monitored over time (Figure 4C). Day 0 treatment with the parent construct yielded robust silencing at all time points, with ~60% knockdown persisting at day 25. The Sense w/o PS variant maintained silencing comparable to the parent construct. In contrast, compounds missing PS linkages on either the 3′‐or 5′‐end of the antisense strand (3′ w/o PS and 5′ w/o PS variants) showed reduced durability, each achieving only ~10% knockdown at day 25. The 3′ w/o PS variant exhibited consistently lower performance across all time points, while the 5′ w/o PS variant retained strong initial potency but declined after prolonged culture. The dual‐modification‐loss variant (3′ + 5′ w/o PS) performed worst, showing both the poor initial potency of the 3′ w/o PS and poor durability of the 5′ w/o PS, with no detectable silencing remaining at day 25. Notably, total siRNA levels in the spheroids were similar across all variants (Figure S3A), peaking at day 7 and steadily declining thereafter. No reduction in cell viability was observed under any condition (Figure S3B).

For in vivo evaluation and comparison to the spheroids model, the parent construct and the 3′ w/o PS were tested in mice. Animals received 25 or 50 mg/kg subcutaneously, and AHSA1 silencing and hepatic siRNA concentrations were measured after 7 days (Figures 4D and S3B). The parent construct achieved ~50% silencing with clear dose‐dependent liver siRNA accumulation. The 3′ w/o PS variant produced only a slight knockdown at the highest dose and showed no dose‐dependent liver siRNA accumulation.

To assess intrinsic silencing potency, all constructs were transfected into HepG2 cells using lipofectamine, and AHSA1 expression was measured at 24 h (Figure S4, Table S1), a time point minimally affected by proliferation‐driven dilution. No substantial differences in silencing potency were observed across constructs under these conditions.

## 4 Discussion

RNA interference (RNAi)‐based therapeutics represent a promising new class of medicine, attracting significant interest across various fields. However, their development is dependent on the quality of preclinical models used to assess safety and efficiency. The current gold standard human in vitro models lack the necessary complexity and longevity to study the long‐term effects of these durable siRNA compounds. Animal models used for the validation of RNA‐based therapeutics need to carry the human transcript of the siRNA target and carry high costs. Moreover, they often fail to accurately mimic human physiology and we therefore developed a long‐term primary human liver spheroid model. This model enables the monitoring and screening of siRNA uptake, potency, and durability, and thus could function as a valuable screening platform in drug development as it has the potential to detect poor uptake and filter out compounds that show good initial potency but lack long‐term durability. This could reduce the reliance on and the number of animals required and accelerate drug development. The relevance of this model is underscored by the fact that all six FDA‐approved RNAi therapeutics target the liver [1].

We found that the ASGR1 protein was uniformly present throughout spheroid cultures. However, following spheroid formation, we observed variability in expression intensity among hepatocytes. This heterogeneity was consistent with ASGR1 mRNA expression patterns and likely reflects zonal specialization within the liver [19, 21]. It appears plausible that this in part explains the more complete silencing of the AHSA1 mRNA expression when cells are treated with GalNAc‐conjugated siRNA on day 0, prior to spheroid formation, compared to culture day 7, when spheroids are formed, and the cells are differentiated. Cultures treated with GalNAc‐conjugated siRNA exhibited rapid and efficient silencing of the target gene AHSA1, whereas non‐conjugated siRNA failed to induce silencing without lipofectamine treatment. This confirms that the GalNAc moiety as described is essential for cellular uptake and subsequent gene silencing and that this is maintained in the spheroid cultures.

The efficiency of siRNA mediated silencing was highly dependent on the timing of treatment: Early treatment, before spheroid formation (day 0), resulted in significantly greater silencing efficiency than treatment at day 7 after spheroid formation. Thus, a ~25% difference in AHSA1 expression between day 0‐ and day 7‐treated spheroids persisted for 5 weeks (Figure 2D). This variation is likely due to reduced accessibility of hepatocytes in mature spheroids. Supporting this, single hepatocytes exposed to fluo‐siRNA showed widespread uptake after 4 h, whereas in spheroids, the signal was predominantly detected in outer‐layer cells (Figure 3A). These findings show that treatment of the cultures before spheroid formation (day 0) results in a more efficient silencing and a larger therapeutic window with more discriminatory power for screening purposes. However, both here (Figure 1) and in previous work it was shown that the spheroids first dedifferentiate and then mature after aggregation and spheroid formation [14, 19]. This model allows for a flexible time of treatment and depending on the target it could be more predictive to treat fully formed spheroids, when they more closely recapitulate the in vivo liver.

The initial efficiency of silencing was essentially independent of the siRNA concentrations used in our experiments (Figure 2C), further suggesting that accessibility is a key factor. Additionally, siRNA levels in spheroids treated on day 0 (before spheroid formation) were ~10‐fold higher compared to those treated on day 7 (after spheroid formation) (Figure 2D). Lipofectamine‐transfected spheroids had higher siRNA levels (Figure S1D), suggesting that ASGPR‐mediated uptake and the subsequent endosomal escape is rate‐limiting. Whereas transfection of siRNA using lipofectamine follows a different silencing kinetic, and the siRNA is retained in the developing lysosome and is therefore not limited by the process of endosomal escape.

Although siRNA distribution was more uniform following day 0 treatment (before spheroid formation), long‐term tracking of fluo‐siRNA over 7 days post‐treatment revealed no major differences in fluorescent signal intensity between day 0‐ and day 7‐treated spheroids (Figure 3B). However, heterogeneity within the hepatocyte population may also contribute to the observed differences in silencing. Fluo‐siRNA accumulated less in CYP3A4+ pericentral hepatocytes, which may reflect inherent zonal specialization within the liver (Figure 3C) [21]. Adjacent liver tissue sections stained for CYP3A4 and ASGR1 suggest that hepatocytes surrounding the central vein (high CYP3A4 expression) have lower ASGR1 expression (Figure S2), which could explain the lower siRNA uptake in the CYP3A4+ hepatocytes. ASGR1 expression is known to be altered in liver diseases such as fibrosis, cirrhosis, and hepatocellular carcinoma, indicating additional regulatory mechanisms that warrant further investigation [22, 23].

Screening of siRNA constructs with varying numbers and positions of PS modifications in human liver spheroids revealed marked differences in silencing durability. Removal of PS modifications from the sense strand had no measurable effect on chemical stability, potency, or durability. In contrast, loss of PS modifications from the antisense strand affected the chemical stability and durability of the siRNA. Particularly, the 3′ w/o PS variant performed poorly in spheroid cultures with low initial potency and durability. The 5′ w/o PS variant retained initial potency but failed to maintain silencing during extended culture. This was not reflected in the short‐term metabolic stability assay, which showed a substantial loss of stability in the 5′ w/o PS variant. This suggests the process of uptake, lysosomal escape, RISC loading, silencing, and degradation captured in the spheroids covers the more dynamic intracellular environment. These results show that there is a difference between the metabolic stability of the 5′ w/o PS variant and the performance in a long‐term in vitro model. This is further emphasized by the lack of difference in the siRNA content of the different variants (Figure S3A). This could indicate a lack of endosomal escape or RISC loading. Constructs lacking PS modifications at both antisense strand termini exhibited severe silencing loss at all time points, underscoring the critical role of these positions in maintaining stability and long‐term performance [24].

These findings indicate that antisense strand PS modifications protect siRNA from nuclease degradation and may promote retention within the RNA‐induced silencing complex (RISC) over extended periods [25]. Notably, such differences were clearly resolved using the liver spheroid model. The loss of 5′ w/o PS silencing efficiency after prolonged culture highlights the need for long‐term culture models. In vivo results mirrored spheroid data: the 3′ w/o PS variant showed markedly reduced AHSA1 silencing and no evidence of dose‐dependent liver accumulation to the parent construct.

A possible limitation of this study is that only AHSA1 was used as a target gene. This gene was chosen as it is stably and broadly expressed in various cell types. Other target genes that have varying or inducible expression levels or have higher mRNA turnover rates could present with a different silencing pattern. However, providing the stability of the siRNA and the reservoir effects of the endo‐lysosomes, the differences between different siRNA variants will likely still be detectable. Additionally, in this study, donors with broadly different characteristics were used with no discernible loss in silencing potency for AHSA1. However, depending on the specific target, the donor characteristics could influence the response and should be well characterized [26, 27].

Overall, human liver spheroids captured the full spectrum of GalNAc–siRNA uptake, potency, and durability. This model enabled clear discrimination between the durability of distinct siRNA backbones, which were exemplarily validated in vivo in mice.

## 5 Implications for Preclinical siRNA Therapeutic Development

The success of siRNA therapeutics relies on the quality of preclinical models. There is an increasing need for long‐term human‐based systems to evaluate candidate siRNA molecules before advancing too costly in vivo studies.

Our findings demonstrate that human liver spheroids provide a robust and scalable model for evaluating long‐acting GalNAc‐conjugated siRNA constructs. Their long‐term stability, functional hepatocyte maintenance, and ease of treatment make them a valuable tool for screening siRNA variants in early‐stage RNAi therapeutic development and could reduce the number of animals required during RNAi development.

## Author Contributions

S.v.R., M.I.‐S., C.G., and T.E. wrote the manuscript; S.v.R, M.I.‐S., C.G., and T.E. designed the research; S.v.R, G.‐J.S., Å.N., L.S., V.E., and C.C. performed the research; S.v.R. and G.‐J.S. analyzed the data.

## Funding

This study was funded by Novartis AG, Basel, Switzerland, by the ERC PoC grant agreement 101123215 and by the Swedish Brain Foundation (grant FO2023‐0139).

## Conflicts of Interest

Magnus Ingelman‐Sundberg is a co‐founder and co‐owner of HepaPredict AB.

## Supporting information


**Figure S1:** Spheroid ATP concentrations following siRNA treatment. (A) Schematic overview of the GalNAc structure used. (B) ATP measurement of liver spheroids on day 14 treated with different concentrations of the parent construct. (C) ATP measurement of liver spheroids on day 7 and day 33 of untreated and parent construct treated. (D) AHSA1 gene expression in long‐term cultures of liver spheroids transfected with non‐conjugated siRNA (‐GalNAc) using lipofectamine (100 nM) on day 0. Overlay siRNA concentration measured in transfected spheroids (*n* = 1).

**Figure S2:** CYP3A4 and ASGR1 staining on adjacent liver sections. Illustrative fluorescent images of cryosections of adjacent healthy liver tissue. CYP3A4 was used as a marker to determine the area surrounding the central vein. The adjacent section was stained for ASGR1 and compared to the CYP3A4 staining. ASGR1 staining was higher in the areas surrounding the portal area, where CYP3A4 signal was absent.

**Figure S3:** Viability and siRNA concentration of PS modified siRNA screening. (A) siRNA measurements on day 7, 18 and 25 of spheroids treated with different siRNA constructs (Free uptake 100 nM; *n* = 3). (B) ATP measurement of liver spheroids on day 7 and day 25 treated with different siRNA variants. (C) siRNA measurement of mice livers treated with either the parent construct or the lowest performing variant in spheroids (3′ w/o PS) at 25 or 50 mg/kg (*n* = 3 animals per group).

**Figure S4:** Lipofectamine transfection of HepG2 cells. AHSA1 mRNA expression in HepG2 cells 24 h following lipofectamine transfection with different concentrations of siRNA variants (*n* = 3).

**Table S1:** IC50 and max silencing of siRNA constructs in HepG2.
