# Supplementary material for: A Long‐Term Human Liver Spheroid Model for Assessing Silencing and Durability of GalNAc‐Conjugated siRNAs
Source: Clin Transl Sci. 2026 Apr 8;19(4):e70536. doi: 10.1111/cts.70536 (PMC13059674; doi:10.1111/cts.70536)
Supplement: Supplementary file 1 — Figure S1: Spheroid ATP concentrations following siRNA treatment. (A) Schematic overview of the GalNAc structure used. (B) ATP measurement of liver spheroids on day 14 treated with different concentrations of the parent construct. (C) ATP measurement of liver spheroids on day 7 and day 33 of untreated and parent construct treated. (D) AHSA1 gene expression in long‐term cultures of liver spheroids transfected with non‐conjugated siRNA (‐GalNAc) using lipofectamine (100 nM) on day 0. Overlay siRNA concentration measured in transfected spheroids (n = 1). [file CTS-19-e70536-s004.pdf]

Figure S1

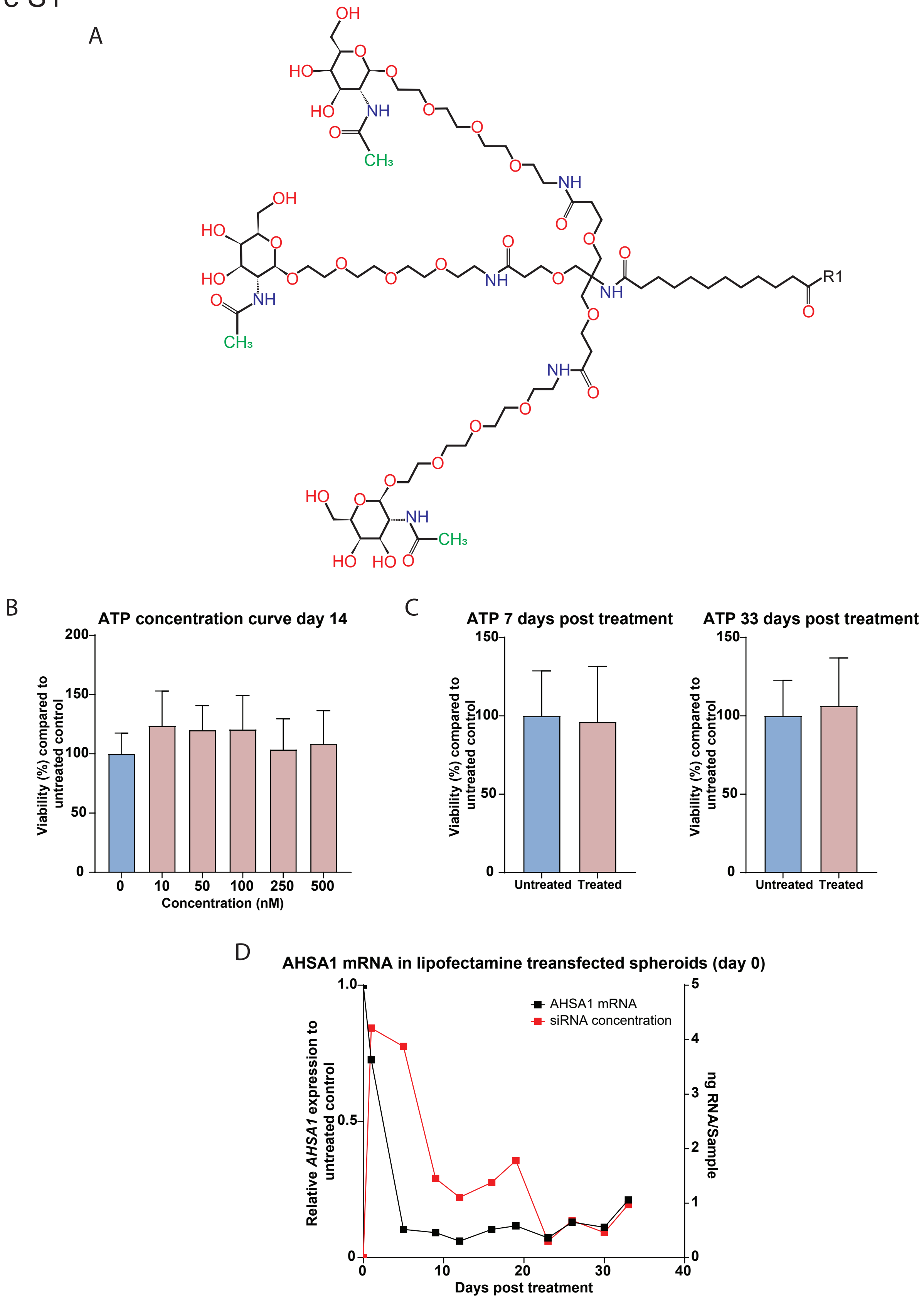

Figure S1. **Spheroid ATP concentrations following siRNA treatment**. **A:** Schematic overview of the GalNAc structure used. **B:** ATP measurement of liver spheroids on day 14 treated with different concentrations of the parent construct. **C:** ATP measurement of liver spheroids on day 7 and day 33 of untreated and parent construct treated. **D:** AHSA1 gene expression in long-term cultures of liver spheroids transfected with non-conjugated siRNA (-GalNAc) using lipofectamine (100 nM) on day 0. Overlay siRNA concentration measured in transfected spheroids. (n = 1).
