# Supplementary material for: A Long‐Term Human Liver Spheroid Model for Assessing Silencing and Durability of GalNAc‐Conjugated siRNAs
Source: Clin Transl Sci. 2026 Apr 8;19(4):e70536. doi: 10.1111/cts.70536 (PMC13059674; doi:10.1111/cts.70536)
Supplement: Supplementary file 2 — Figure S2: CYP3A4 and ASGR1 staining on adjacent liver sections. Illustrative fluorescent images of cryosections of adjacent healthy liver tissue. CYP3A4 was used as a marker to determine the area surrounding the central vein. The adjacent section was stained for ASGR1 and compared to the CYP3A4 staining. ASGR1 staining was higher in the areas surrounding the portal area, where CYP3A4 signal was absent. [file CTS-19-e70536-s003.pdf]

Figure S2

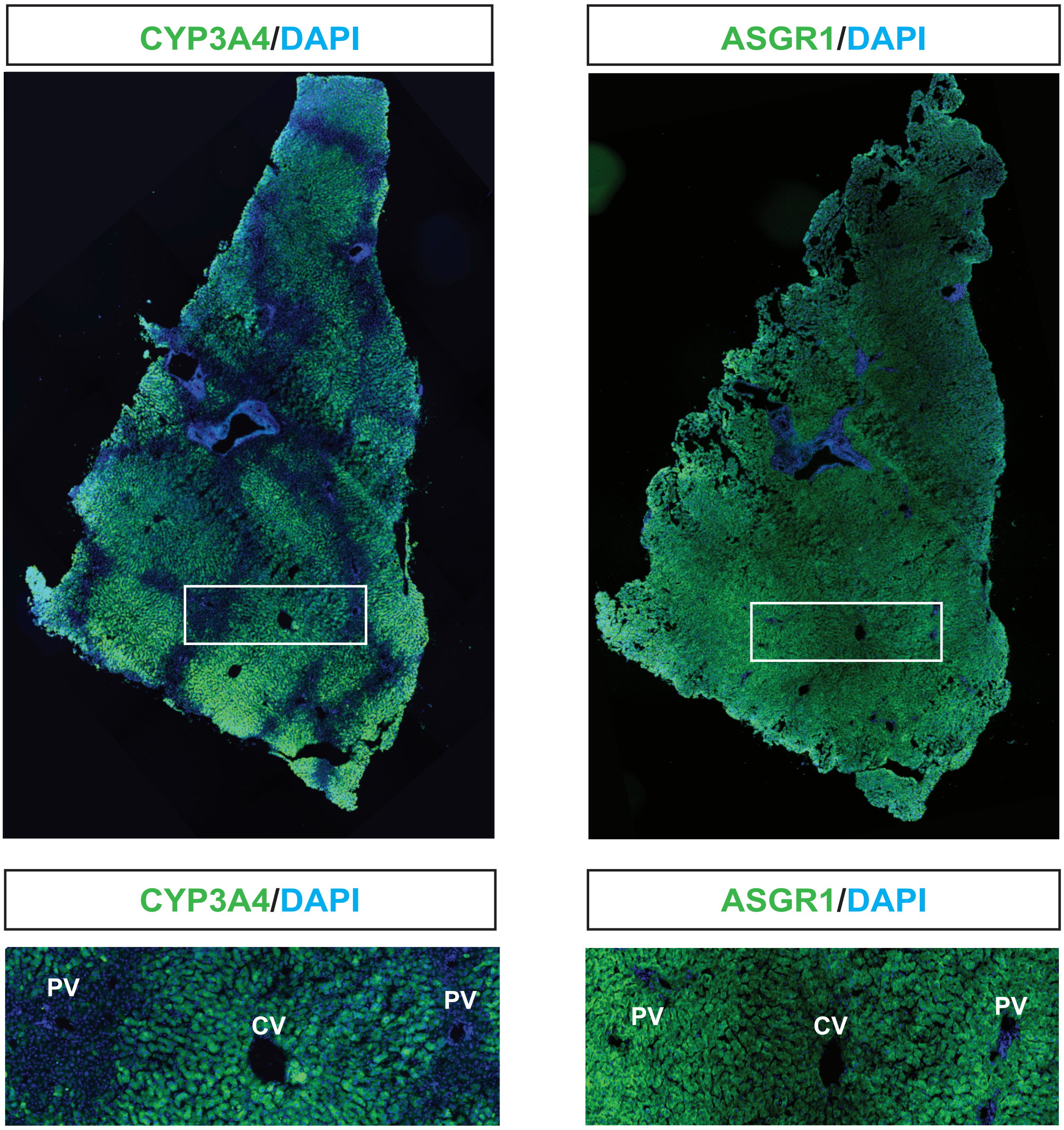

Figure S2. CYP3A4 and ASGR1 staining on adjacent liver sections. Illustrative fluorescent images of cryosections of adjacent healthy liver tissue. CYP3A4 was used as a marker to determine the area surrounding the central vein. The adjacent section was stained for ASGR1 and compared to the CYP3A4 staining. ASGR1 staining was higher in the areas surrounding the portal area, where CYP3A4 signal was absent.
