# Supplementary material for: A Long‐Term Human Liver Spheroid Model for Assessing Silencing and Durability of GalNAc‐Conjugated siRNAs
Source: Clin Transl Sci. 2026 Apr 8;19(4):e70536. doi: 10.1111/cts.70536 (PMC13059674; doi:10.1111/cts.70536)
Supplement: Supplementary file 3 — Figure S3: Viability and siRNA concentration of PS modified siRNA screening. (A) siRNA measurements on day 7, 18 and 25 of spheroids treated with different siRNA constructs (Free uptake 100 nM; n = 3). (B) ATP measurement of liver spheroids on day 7 and day 25 treated with different siRNA variants. (C) siRNA measurement of mice livers treated with either the parent construct or the lowest performing variant in spheroids (3′ w/o PS) at 25 or 50 mg/kg (n = 3 animals per group). [file CTS-19-e70536-s001.pdf]

Figure S3

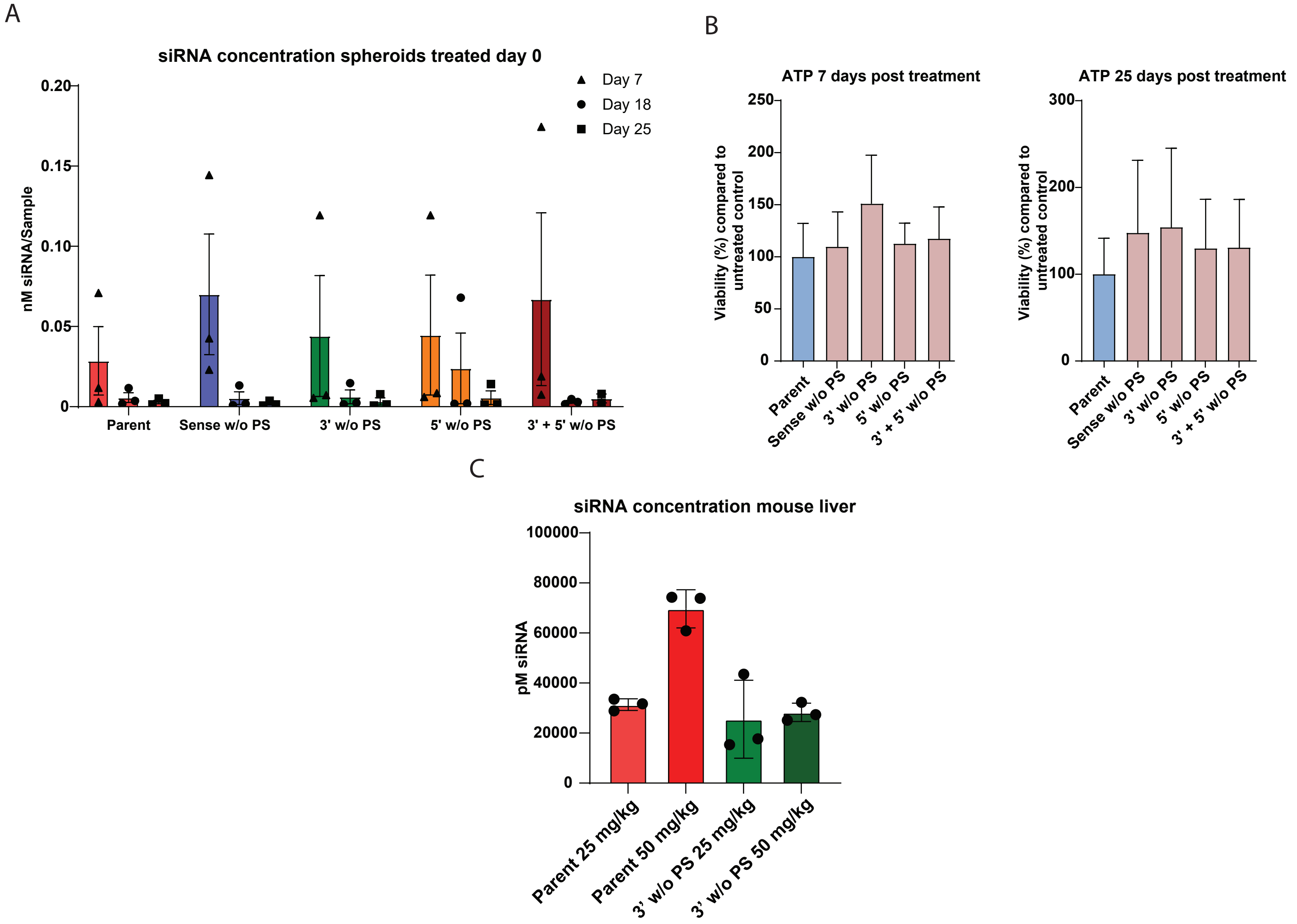

Figure S3. Viability and siRNA concentration of PS modified siRNA screening. A: siRNA measurements on day 7, 18 and 25 of spheroids treated with different siRNA constructs (Free uptake 100 nM; n = 3). **B:** ATP measurement of liver spheroids on day 7 and day 25 treated with different siRNA variants. **C:** siRNA measurement of mice livers treated with either the parent construct or the lowest-performing variant in spheroids (3′ w/o PS) at 25 or 50 mg/kg (n = 3 animals per group).
