# Supplementary material for: A Long‐Term Human Liver Spheroid Model for Assessing Silencing and Durability of GalNAc‐Conjugated siRNAs
Source: Clin Transl Sci. 2026 Apr 8;19(4):e70536. doi: 10.1111/cts.70536 (PMC13059674; doi:10.1111/cts.70536)
Supplement: Supplementary file 4 — Figure S4: Lipofectamine transfection of HepG2 cells. AHSA1 mRNA expression in HepG2 cells 24 h following lipofectamine transfection with different concentrations of siRNA variants (n = 3). [file CTS-19-e70536-s005.pdf]

Figure S4

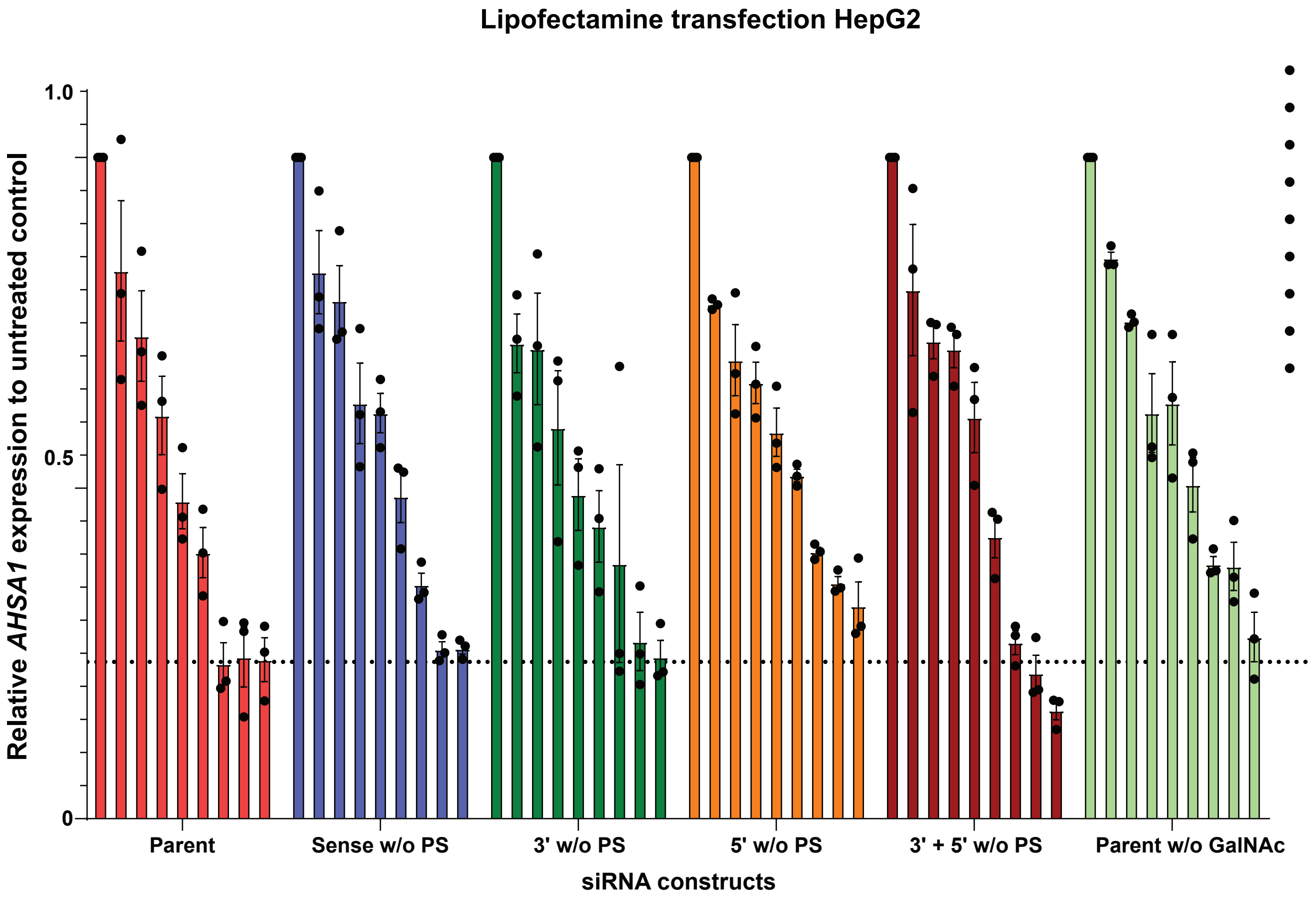

Figure S4. **Lipofectamine transfection of HepG2 cells**. AHSA1 mRNA expression in HepG2 cells 24 hours following lipofectamine transfection with different concentrations of siRNA variants (n = 3).
