# Supplementary material for: A Long‐Term Human Liver Spheroid Model for Assessing Silencing and Durability of GalNAc‐Conjugated siRNAs
Source: Clin Transl Sci. 2026 Apr 8;19(4):e70536. doi: 10.1111/cts.70536 (PMC13059674; doi:10.1111/cts.70536)
Supplement: Supplementary file 5 — Table S1: IC50 and max silencing of siRNA constructs in HepG2. [file CTS-19-e70536-s002.docx]

**Table S1: IC50 and max silencing of siRNA constructs in HepG2**

| siRNA construct | IC50 | Max Silencing at 50 nM |
| --- | --- | --- |
| Parent | 1,88 nM | 0,240 ± 0,0573 |
| Sense w/o PS | 3,485 nM | 0,257 ± 0,0148 |
| 3' w/o PS | 2,963 nM | 0,244 ± 0,0440 |
| 5' w/o PS | 2,963 nM | 0,322 ± 0,0629 |
| 3' + 5' w/o PS | 4,038 nM | 0,275 ± 0,0650 |
| Parent w/o GalNAc | 3,547 nM | 0,164 ± 0,0248 |
